# *Borrelia* Infections in Ageing Ticks: Relationship with Morphometric Age Ratio in Field-Collected *Ixodes ricinus* Nymphs

**DOI:** 10.3390/microorganisms10010166

**Published:** 2022-01-13

**Authors:** Andrea Springer, Daniela Jordan, Antje Glass, Olaf Kahl, Volker Fingerle, Philipp Girl, Lidia Chitimia-Dobler, Christina Strube

**Affiliations:** 1 Institute for Parasitology, Centre for Infection Medicine, University of Veterinary Medicine Hannover, 30559 Hanover, Germany; andrea.springer@tiho-hannover.de (A.S.); daniela.jordan@tiho-hannover.de (D.J.); antje.glass@tiho-hannover.de (A.G.); 2 Tick-Radar GmbH, 10555 Berlin, Germany; olaf.kahl@berlin.de; 3 National Reference Center for Borrelia, Bavarian Health and Food Safety Authority, 85764 Oberschleissheim, Germany; volker.fingerle@lgl.bayern.de; 4 Bundeswehr Institute of Microbiology, 80937 Munich, Germany; philippgirl@bundeswehr.org (P.G.); lydiachitimia@gmail.com (L.C.-D.)

**Keywords:** Lyme borreliosis, tick-borne pathogens, *Borrelia burgdorferi* s.l., *Borrelia miyamotoi*, biological age, morphometric age ratio

## Abstract

In Europe, *Ixodes ricinus* plays a major role as a vector of *Borrelia burgdorferi* sensu lato (s.l.) spirochaetes, the causative agents of Lyme borreliosis, among other pathogens. In unfed ticks, *Borrelia* spirochaetes experience prolonged nutrient restriction. However, only few studies exist with regard to *Borrelia* infections in unfed ticks of different physiological ages. Changing body dimensions of unfed ticks, due to the consumption of energy reserves, allow physiological age estimation. The present study investigated the relationship of morphometric age with *Borrelia* prevalence and spirochaete load in 1882 questing *I. ricinus* nymphs, collected at two different locations in northern Germany in 2020. In addition, *Borrelia* species composition was investigated by employing a reverse line blot (RLB) probe panel suitable for the detection of ten different *B. burgdorferi* s.l. species, as well as the relapsing-fever spirochaete *B. miyamotoi*. Overall, *Borrelia* prevalence was 25.8% (485/1882). Whilst there was no statistically significant difference in *Borrelia* prevalence between the different morphometric age groups, *Borrelia* infection intensity as determined by probe-based quantitative real-time PCR significantly declined with increasing morphometric age. *Borrelia* species differentiation by RLB was successful in 29.5% of positive ticks, and revealed *B. afzelii* as the dominating species (65.0% of the differentiated infections). Additionally, *B. garinii*, *B. valaisiana*, *B. burgdorferi* sensu stricto, *B. spielmanii*, and *B. miyamotoi* were detected.

## 1. Introduction

Ticks of the *Ixodes ricinus* species complex, particularly *I. ricinus* in Europe, *I. persulcatus* in Asia, and *I. scapularis* in North America, are of major public health importance due to their vector function for various pathogens, including *Borrelia burgdorferi* sensu lato (s.l.) spirochaetes, the causative agents of Lyme borreliosis [1]. They are exophilic three-host ticks, which take one blood meal per life stage (larva, nymph, adult) and complete their life cycle within approximately three to six years [2]. Most of this time is spent off-host, where the tick faces a trade-off between the need to find a host by questing on vegetation, and maximizing survival by conserving energy and avoiding the risk of desiccation [2]. In an unfed tick, extracellular pathogens, such as *Borrelia* spirochaetes, experience prolonged nutrient restriction and need to adapt their metabolism accordingly [3]. However, only few studies exist with regard to *Borrelia* infections in unfed ticks of different physiological ages. For *B. afzelii*, one of the most prevalent members of the *B. burgdorferi* s.l. complex in Europe, a decline in unfed nymphal spirochaete load over time has been shown under laboratory conditions [4], possibly affecting the tick-to-host transmission efficiency. However, associations of tick physiological age with pathogen infection or pathogen load in field-collected ticks may also be influenced by the pathogen’s ability to manipulate tick physiology and behavior to promote their own transmission. For example, *B. afzelii* infections enhance tick survival under desiccating conditions [5], so that a higher *Borrelia* prevalence among older ticks might be expected.

In *I. ricinus* populations of Central Europe, the common seasonal pattern of tick activity includes a peak in spring and, in some years, a second, lower peak in autumn, while moulting occurs during the warmest months of the year [2]. This pattern arises as ticks respond to ambient conditions, such as temperature and relative humidity. Moreover, they have evolved developmental strategies to avoid questing at unfavorable times of the year, as extensively reviewed by Gray et al. [2]. These strategies include either a developmental diapause or a behavioural quiescence during winter. The former occurs with regard to *I. ricinus* eggs, engorged larvae, and nymphs, while the latter pertains to unfed nymphs and adults. As a result, most unfed nymphal ticks are expected to become active approximately one year after their larval blood meal, while a minority of nymphs may start questing in autumn of the same year if molting occurred early during the summer. Due to the flexibility of these strategies, considerable variation of this basic pattern may occur, resulting in the simultaneous questing activity of ticks from different generational cohorts during the same season [2].

As off-host ticks consume their energy reserves, particularly stored in the form of lipids [6,7], their body dimensions change over time [7]. Thus, measurement of the soft components of the tick body, i.e., the alloscutum, in relation to the non-changing, sclerotized scutum can be used to derive the so-called morphometric age ratio, an estimate of the physiological age of unfed ticks [8]. Based on this method, a previous study on field-collected *I. persulcatus* suggested that there may be a negative relationship between tick physiological age and the intensity of infection with *B. burgdorferi* s.l.; however, only 131 ticks were examined, of which only 46 were infected [8].

The present study investigated the relationship of morphometric age with *Borrelia* infection status and spirochaete load in 1882 questing *I. ricinus* nymphs, collected from March to October 2020 at two different locations in northern Germany. Apart from the abovementioned studies, little information is available regarding the development of *Borrelia* infections in unfed ticks over time, particularly with regard to the different species of the *B. burgdorferi* s.l. complex present in Europe, which vary in their genetic content, reservoir association, replication dynamics during blood-feeding, pathogenic potential, and clinical manifestation [9]. In addition, the little-studied relapsing-fever spirochaete *B. miyamotoi* co-circulates with *B. burgdorferi* s.l., using the same tick vectors [10]. Therefore, the present study also assessed the *Borrelia* species composition in the collected ticks, employing a reverse line blot (RLB) probe panel suitable for the detection of mono- and co-infections with ten different *B. burgdorferi* s.l. species relevant in Europe, as well as *B. miyamotoi* [11,12].

## 2. Materials and Methods

### 2.1. Tick Collection and Measurement

Nymphal ticks were collected by the flagging method at two locations in northern Germany from March to October 2020: in a peri-urban mixed forest in the region of Hanover, federal state of Lower Saxony, and in a mixed deciduous-coniferous forest close to Neustrelitz in the federal state of Mecklenburg-Western Pomerania. The intended sample size was 150 *I. ricinus* nymphs per month and location.

Ticks were morphologically identified to species level based on phenotypic keys [13] and measured using a Keyence VHX-900F digital microscope (Itasca, IL, USA) when still alive to determine the ratio of the alloscutal/scutal index (morphometric age ratio) according to Uspensky et al. [8]:

((BL × BW) − (SL × SW))/(SL × SW),

where SL is the scutal length from the anterior edge to the posterior tip of the scutum at the midline, SW is the scutal width at the widest point of the scutum, BL is the body length from the anterior edge of the scutum to the posterior tip of the opisthosoma at the midline, and BW is body width at the widest point after the posterior tip of the scutum. As the alloscutal index ((BL × BW) − (SL × SW)) gradually declines during tick starvation while the scutal index (SL × SW) remains constant, their ratio gradually decreases in value as the tick ages and can be used for estimation of tick physiological age [7,8]. Age category definitions are shown in Table 1. After morphometric measurements, ticks were stored at −20 °C until further analyses.

### 2.2. DNA Isolation and Quantitative Real-Time PCR

DNA was isolated from individual ticks using the NucleoSpin 8 Blood Kit (Macherey-Nagel GmbH & Co KG, Düren, Germany) as previously described [15]. A primer-probe combination targeting the *Borrelia* 5S-23S ribosomal RNA intergenic spacer (IGS) region [16] was employed for *Borrelia* detection by quantitative real-time PCR, with the previously published reaction set-up and thermoprofile [17,18]. A part of the *Ixodes* internal transcribed spacer 2 (ITS2) region was co-amplified to verify DNA isolation success [16,19]. All reactions were performed in duplicates and included serial plasmid standards (10^0^−10^6^ copies) as well as a negative control. 

### 2.3. Reverse Line Blot

*Borrelia*-positive samples were subjected to a conventional PCR targeting a fragment of the 5S–23S IGS by use of primers B5S-Bor, 23SBor, and BMiya-For [12,20], followed by *Borrelia* species differentiation by RLB using established protocols [11,12]. In the present study, an additional probe for *B. spielmanii* detection, SPiNE3T (5′-[AmC6]GAATAAGTCATTTAAATAACATA-3′), was included due to sequence variation between different *B. spielmanii* strains (Table 2). *Borrelia* strains included as positive controls in the RLB are shown in Table 2. The method allows unambiguous discrimination of *B. afzelii*, *B. bissettiae*, *B. lusitaniae*, *B. spielmanii*, *B. valaisiana*, *B. kurtenbachii*, and *B. miyamotoi*. Furthermore, *B. garinii* and *B. bavariensis* as well as *B. burgdorferi* s.s. and *B. carolinensis* are also detected, but Sanger sequencing is required for unambiguous identification due to cross-reactions. Thus, PCR products reacting with probes GA and SS (Table 2) were re-amplified and custom Sanger-sequenced (Microsynth Seqlab, Göttingen, Germany). Obtained sequences were aligned with reference strains using Clone Manager (Version Professional 9, Sci Ed Software, Westminster, CO, USA).

### 2.4. Statistical Analyses

Statistical analyses were conducted in R software v. 4.1.0 [24]. *Borrelia* species identification success by RLB was compared between the two different sampling locations via χ^2^-test. To compare the distribution of *Borrelia* species between sampling locations and tick morphometric age groups, Fisher’s Exact tests were employed.

To assess the effect of tick morphometric age on *Borrelia* prevalence, a generalized linear mixed model (GLMM) with a binomial error structure and logit link function was constructed using the package lmerTest [25], including the categorical variables “morphometric age ratio” and “sampling month” and the continuous variable “*Ixodes* ITS2 copy number,” to control for DNA isolation efficiency as a possible confounder. ITS2 copy numbers were log transformed prior to analysis. “Sampling location” was entered as a random factor.

Furthermore, the relationship of the same variables with the *Borrelia* 5S–23S IGS copy numbers was investigated in *Borrelia*-positive ticks using a linear mixed model (LMM). For this purpose, *Borrelia* 5S–23S IGS copy numbers were log transformed to meet model assumptions. In addition, a linear model (LM) was fitted including only those ticks with identified *B. afzelii* infection and the fixed factors as in the first model. Sampling location was not taken into account, as an initial LMM including this variable as a random factor produced a singular model fit.

Normality and distribution of model residuals against fitted values were assessed graphically. Shapiro–Wilk and D’Agostino’s *K*-squared test were additionally employed to check the normality of model residuals. Full models were compared to null models containing only the random factor using the R function “anova.” 

## 3. Results

### 3.1. Borrelia Prevalence and Species Distribution

Overall, 2008 nymphal ticks were collected; however, ticks with less than 10^4^ *Ixodes* ITS2 copies were excluded from the analysis due to questionable DNA isolation efficiency. Consequently, 1882 ticks were analyzed in total. 

**Figure 1:**
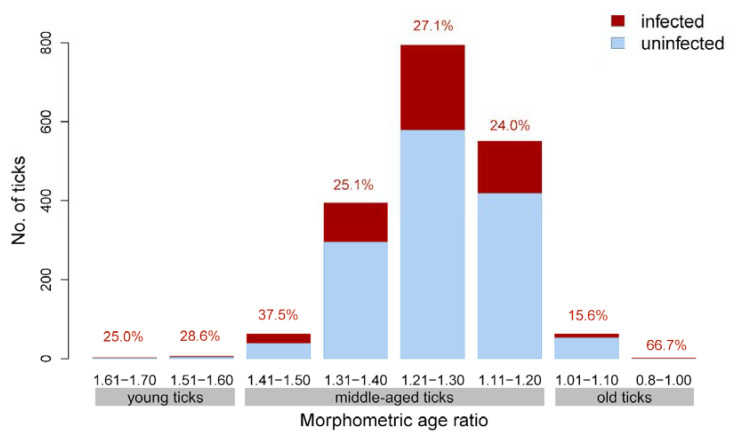
Distribution of the morphometric age ratio and *Borrelia* prevalence in the dataset of 1882 *I. ricinus* ticks. Percentages indicate the *Borrelia* prevalence in each category.

The overall *Borrelia* prevalence was 25.8% (485/1882), with values of 23.4% (249/1063) in Hanover and 28.8% (236/819) in Neustrelitz. Among the eight different morphometric age ratio categories, *Borrelia* prevalence varied between 15.6% (10/64) and 66.7% (2/3) (Figure 1). Monthly prevalence values are shown in Table 3. 

*Borrelia* species differentiation by RLB was successful in 29.5% (143/485) of positive ticks. Differentiation success was dependent on *Borrelia* copy numbers, and amounted to 95.3% (41/43), 61.1% (55/90), 26.1% (31/119), and 6.9% (16/233) in ticks with *Borrelia* 5S–23S IGS copies of ≥10^3^, ≥10^2^ to <10^3^, ≥10 to <10^2^, and <10, respectively. Differentiation success was higher for ticks collected in Neustrelitz than for ticks collected in Hanover (33.9% [80/236] vs. 25.3% [63/249], χ^2^-test, χ^2^ = 3.9, *p* = 0.048), although the distribution of *Borrelia* copy numbers did not differ between the two locations (Wilcoxon rank sum test, W = 28,160, *p* = 0.428). 

*Borrelia afzelii* was the most frequently detected species among the differentiated samples (65.0%, 93/143), followed by *B. garinii/B. bavariensis* and *B. spielmanii* (9.8%, 14/143 each), *B. miyamotoi* (9.1%, 13/143), *B. valaisiana* (7.0%, 10/143), and *B. burgdorferi* s.s./*B. carolinensis* (2.1%, 3/143). Among the *B. spielmanii*-positive samples, 7/14 reacted only with the SpiNE3T probe, but not with the SpiNE3 probe. To verify *B. spielmanii*, four of these samples were sequenced, yielding 5S–23S IGS sequences of 116 bp 100% identical to *B. spielmanii* strains PHap, PMai, and PSigII (100% query cover). Sequencing of the 14 *B. garinii*/*B. bavariensis*-positive samples revealed *B. garinii* in 13 cases, whereas sequencing of one sample was not successful. In all three *B. burgdorferi* s.s./*B. carolinensis*-positive samples, *B. burgdorferi* s.s. was identified by sequencing. 

Coinfections were present in four cases (twice with *B. garinii* and *B. valaisiana*, once with *B. afzelii* and *B. miyamotoi*, and once with *B. afzelii* and *B. spielmanii*). *Borrelia lusitaniae*, *B. kurtenbachii*, *B. bissettiae*, and *B. carolinensis* were not detected.

**Figure 2:**
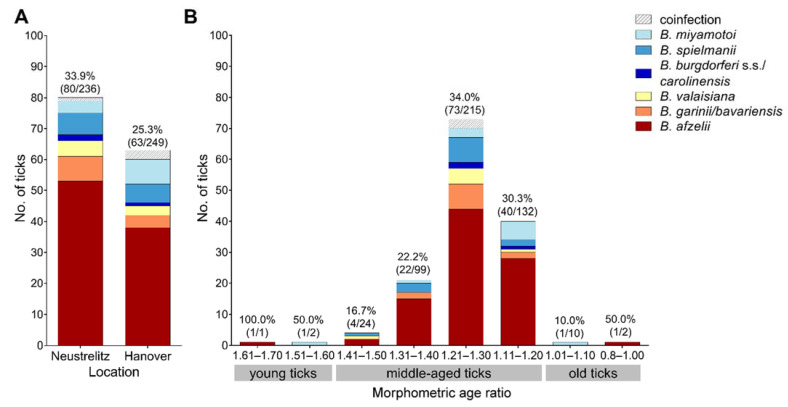
*Borrelia* spp. composition as determined by RLB among the 142 successfully differentiated samples according to (**A**) sampling location and (**B**) tick morphometric age ratio. Percentages above the bars indicate RLB differentiation success in each category. Coinfections consisted of *B. garinii/bavariensis* + *B. valaisiana* (1× in Neustrelitz, and 1× in Hanover), *B. afzelii* + *B. valaisiana* (1× in Hanover), and *B. afzelii* + *B. miyamotoi* (1× in Hanover).

RLB results regarding the two sampling locations as well as the different tick morphometric age groups are shown in Figure 2. The *Borrelia* species composition did not show any significant differences, neither between the two locations (Fisher’s Exact test, *p* = 0.256) nor the morphometric age groups (Fisher’s Exact test, *p* = 0.242).

### 3.2. Relationship of Borrelia Prevalence and Infection Intensity with Tick Morphometric Age

Analysis by GLMM indicated no significant differences in *Borrelia* prevalence with regard to tick morphometric age groups, but there was a seasonal difference with a significantly lower prevalence in May than in March (Table 4). Similarly, no statistically significant association between *Borrelia* prevalence and *Ixodes* ITS2 copy numbers was found.

**Figure 3:**
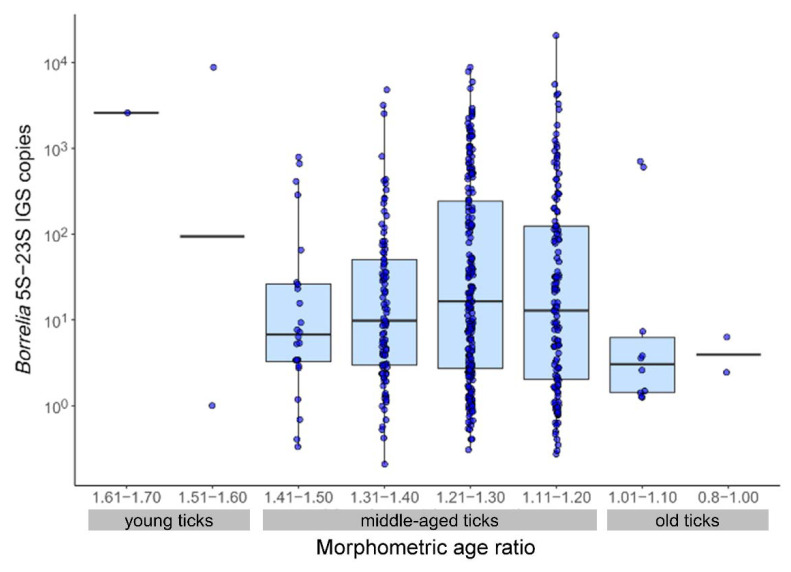
*Borrelia* 5S–23S IGS copies in the different morphometric age groups. Boxplots are shown for groups with *n* ≥ 5, while individual data points are shown in dark blue. The thick line indicates the median.

Among the 485 *Borrelia*-positive ticks, a negative relationship between tick morphometric age group and *Borrelia* copy number was detected, controlling for the effect of the sampling month and *Ixodes* ITS2 copies (i.e., DNA isolation efficiency). In fact, ticks with a low alloscutal/scutal index ratio of 0.80−1.00 harbored significantly less borreliae than the reference age group with the highest alloscutal/scutal index ratio (Figure 3, Table 5). Differences between sampling months and a positive relationship with *Ixodes* ITS2 copy numbers were also detected (Table 5). The distribution of model residuals did not differ from the normality assumption (Shapiro-Wilk-test: W = 0.99, *p* = 0.111; D’Agostino’s *K*-squared test: skew = −0.08, z = −0.71, *p* = 0.480). When only the 92 ticks with identified *B. afzelii* infection were investigated, the same significant difference between morphometric age groups was detected (Table S1), while the number of identified other species was too low for a separate analysis.

## 4. Discussion

Lyme borreliosis is the most frequent tick-borne disease in the northern hemisphere; however, many aspects of the spirochaetes’ biology within the tick vector remain insufficiently understood. The present study showed a decrease in *Borrelia* infection intensity with increasing morphometric age in unfed *I. ricinus* nymphs, similar to earlier observations of Uspensky et al. [8] in field-collected unfed female *I. persulcatus*. The fact that no difference in infection prevalence was noted among morphometric age groups is also consistent with their observations. 

These results may be explained by several mechanisms: First, *Borrelia* loads may decrease over time in ageing ticks under natural conditions. Indeed, under laboratory conditions, an 80% decrease in *B. afzelii* spirochaete load has been demonstrated in unfed *I. ricinus* nymphs over the course of three months [4]. The spirochaetes’ colonization of and persistence in the tick midgut is associated with complex gene-regulatory changes, interactions with tick proteins, e.g., TROSPA, and manipulation of the tick gut microbiota [26]. If these interactions change over time, spirochaete survival might be affected. However, studies that explicitly examine this hypothesis are not yet available.

Second, ticks with high bacterial loads may have a reduced survival probability, so that ticks with a low infection intensity survive for a longer period. Finally, ticks with high bacterial loads may be better at finding a host, which would remove them from the pool of questing ticks earlier than specimens with a low bacterial load. While these possible underlying mechanisms need to be investigated further in experimental studies, the first and the third possibility seem particularly likely in the light of previous research on tick-*Borrelia* interactions, while evidence for the second hypothesis is rather scarce. While a reduced survival of unfed adult *I. ricinus* with extremely high *B. burgdorferi* s.l. loads under desiccating conditions has been shown, the same study found no effect on the survival of infected unfed nymphs and concluded that, in general, *B. burgdorferi* s.l. infection, and in particular *B. afzelii*, rather confers a survival advantage [5]. Another study showed that field-collected unfed *I. ricinus* nymphs infected with various *B. burgdorferi* s.l. species had higher fat reserves relative to body size, regardless of spirochaete load, resulting in enhanced desiccation tolerance [27]. Resistance to desiccation might influence questing success, as ticks may be able to continue questing for longer periods before having to return to the leaf litter to maintain their water balance. Accordingly, *Borrelia*-infected nymphal *I. scapularis* and adult *I. persulcatus* showed greater questing heights [28,29] and infected unfed *I. ricinus* nymphs as well as *I. persulcatus* females moved less than non-infected ticks in experimental studies [30,31]. Although a relationship with spirochaete load has not yet been shown, it may be hypothesized that the manipulative effects of borreliae are stronger in ticks with higher infection intensities. 

Unfortunately, species differentiation success of *Borrelia*-positive samples was rather low in comparison to previous studies using the same RLB protocol [11,32]. This is most likely due to the fact that only nymphs were investigated, which commonly harbor lower *Borrelia* loads than adult ticks [33,34]. Previous studies have shown that the sensitivity of the RLB depends on *Borrelia* 5S–23S IGS copy numbers [12,32]. This was also obvious in the present study, with similar detection rates in the different copy number categories as compared to previous investigations [12,32]. Analyzing adult female ticks in future studies may therefore be more promising with regard to assessing the effect of tick age on different *Borrelia* species, and to compare the results of the current study in general, although it may be challenging to collect a comparable number of adult ticks in the field.

*Borrelia afzelii* was the most frequently detected species, similar to other studies from northern Germany, although the comparably low detection rate of *B. garinii*/*B. bavariensis* was unexpected [12,32,35]. However, a similar *B. burgdorferi* s.l. species distribution as detected in the present study has been previously reported from the Netherlands [36]. 

When only ticks with *B. afzelii* infection as determined by RLB were included in the model investigating the relationship of 5S–23S IGS copy numbers with tick morphometric age, the same copy number decline in older ticks was observed as in the whole dataset. In contrast, it was not possible to investigate whether this was also true for infections with other *B. burgdorferi* s.l. species due to the relatively low number of successfully identified infections. 

Notably, inclusion of a modified *B. spielmanii*-probe, SpiNe3T, enabled detection of *B. spielmanii* in several samples, which did not react with SpiNE3, despite the fact that both probes differ only by one nucleotide. Therefore, this *B. spielmanii* strain variation should be taken into account in future RLB studies.

No significant difference in *Borrelia* species distribution was evident with regard to the different age groups, although the analysis was limited by the fact that only few ticks with a morphometric age ratio > 1.50 and <1.10 were collected, corresponding to Balashov’s age groups II (young ticks) and IV (old ticks) [8], while most ticks were assigned to group III (middle-aged ticks). Further studies based on equal sample sizes of the different age groups would be ideal to confirm the present observation of lower spirochaete loads in older ticks, and to shed more light on *Borrelia* spp. diversity among the different age groups. However, a sufficient sample size of young/old ticks may be difficult to obtain from the field, so that using laboratory-reared ticks of known age may be required. Furthermore, the morphometric age ratio correlates with tick physiologic age, but is not a perfect estimate, as it may be influenced by the tick’s hydration status [7]. Here, the morphometric age ratio was used as a fast and easily obtained estimate of physiological age, while the determination of the tick’s complete energy budget, including levels of glycogen, carbohydrates, proteins, and stored lipids can be considered the gold standard [37]. In future studies, these more refined methods of determining physiological age can be employed to follow up on the potential mechanisms underlying the observed association of *Borrelia* load with tick age.

## Figures and Tables

**Table 1:** Tick morphometric age categories used in the current study.

Age Category	Alloscutal/Scutal Index	Age Group According to Balashov [14]
1	1.61−1.70	II (young ticks)
2	1.51−1.60	II (young ticks)
3	1.41−1.50	III (middle-aged ticks)
4	1.31−1.40	III (middle-aged ticks)
5	1.21−1.30	III (middle-aged ticks)
6	1.11−1.20	III (middle-aged ticks)
7	1.01−1.10	IV (old ticks)
8	0.8−1.00	IV (old ticks)

**Table 2:** Probes and positive controls used in the reverse line blot for *Borrelia* species discrimination.

Probe	Target Species	*Borrelia* Strain Used as Positive Control	Probe References
SL2	*B. burgdorferi* s.l.	-	[17]
AF	*B. afzelii*	PBas	[21]
GA	*B. garinii*, *B. bavariensis*	PWudII, PBi	[21]
BisNE2	*B. bissettiae*	DN127	[22]
SS	*B. burgdorferi* s.s., *B. carolinensis*	PAbe, SCW-22^T^	[22]
LusiNE2	*B. lusitaniae*	Poti B2	[22]
SpiNE3	*B. spielmanii*	PHap	[23]
SpiNE3T	*B. spielmanii*	PHap	this study
VSNE	*B. valaisiana*	VS116	[23]
BisNE1	*B. kurtenbachii*	25015	[22]
MIYA	*B. miyamotoi*	HT31	[12]

**Table 3:** Monthly *Borrelia* spp. prevalence in questing *I. ricinus* nymphs.

Month	Hanover	Neustrelitz	Total
March	35/151 (23.2%)	48/153 (31.4%)	83/304 (27.3%)
April	46/150 (30.7%)	31/148 (20.9%)	77/298 (25.8%)
May	29/147 (19.7%)	30/149 (20.1%)	59/296 (19.9%)
June	31/147 (21.1%)	44/108 (40.7%)	75/255 (29.4%)
July	40/148 (27.0%)	42/141 (29.8%)	82/289 (28.4%)
August	21/134 (15.7%)	21/63 (33.3%)	42/197 (21.3%)
September	31/118 (26.3%)	3/8 (37.5%)	34/126 (27.0%)
October	16/68 (23.5%)	17/49 (34.7%)	33/117 (28.2%)

**Table 4:** Results of the binomial GLMM investigating the effect of tick morphometric age, sampling month, and *Ixodes* ITS2 copy numbers on *Borrelia* spp. prevalence. The full model was significantly different from a null model containing only the random factor (χ^2^ = 27.13, Df = 15, *p* = 0.028). Significant *p*-values (≤0.050) are shown in bold.

Variable	Estimate	Std. Error	z-Value	*p*-Value
Intercept	−1.92	1.34	−1.43	0.154
Month				
March	Reference	−	−	−
April	−0.07	0.19	−0.36	0.716
May	−0.41	0.20	−2.06	**0.040**
June	0.12	0.19	0.62	0.536
July	0.07	0.19	0.37	0.715
August	−0.06	0.28	−0.21	0.836
September	0.36	0.31	1.15	0.250
October	0.28	0.30	0.91	0.362
Morphometric age ratio				
1.61–1.70	Reference	−	−	−
1.51–1.60	0.16	1.44	0.11	0.910
1.41–1.50	0.51	1.19	0.42	0.672
1.31–1.40	−0.18	1.17	−0.15	0.881
1.21–1.30	−0.01	1.17	−0.01	0.996
1.11–1.20	−0.18	1.17	−0.15	0.878
1.01–1.10	−0.73	1.21	−0.60	0.550
0.80–1.00	1.71	1.69	1.01	0.312
*Ixodes* ITS2 copies ^1^	0.07	0.05	1.49	0.136

^1^ log transformed.

**Table 5:** Results of the LMM investigating the effects of different variables on log-transformed *Borrelia* 5S–23S IGS copy numbers among 485 *Borrelia*-positive ticks. The full model was significantly different from a null model containing only the random factor (χ^2^ = 91.82, Df = 15, *p* < 0.001). Significant *p*-values (≤0.050) are shown in bold.

Variable	Estimate	Std. Error	Df	t-Value	*p*-Value
Intercept	2.25	2.76	466.20	0.81	0.416
Month					
March	Reference	−	−	−	−
April	−0.45	0.38	439.99	−1.18	0.239
May	1.02	0.41	468.98	2.50	**0.013**
June	1.71	0.38	468.08	4.53	**<0.001**
July	2.16	0.38	468.61	5.70	**<0.001**
August	0.79	0.59	416.62	1.33	0.184
September	1.83	0.63	177.98	2.89	**0.004**
October	1.27	0.64	441.67	1.99	**0.048**
Morphometric age ratio					
1.61−1.70	Reference	−	−	−	−
1.51−1.60	−0.88	2.88	468.13	−0.31	0.760
1.41−1.50	−4.50	2.39	468.58	−1.88	0.060
1.31−1.40	−3.78	2.35	468.97	−1.61	0.109
1.21−1.30	−3.41	2.35	468.96	−1.45	0.147
1.11−1.20	−3.85	2.35	468.96	−1.64	0.102
1.01−1.10	−4.45	2.45	468.96	−1.81	0.070
0.80−1.00	−6.48	2.85	468.71	−2.27	**0.023**
*Ixodes* ITS2 copies ^1^	0.25	0.10	361.62	2.52	**0.012**

^1^ log transformed.

## Data Availability

Data supporting reported results are contained within the article.

## Supplementary Materials

The following are available online at https://www.mdpi.com/article/10.3390/microorganisms10010166/s1, Table S1: Results of the LMM investigating the effect different variables on log-transformed *Borrelia* 5S–23S IGS copy numbers among 92 *B. afzelii*-positive ticks.
