# Supplementary material for: Borrelia Infections in Ageing Ticks: Relationship with Morphometric Age Ratio in Field-Collected Ixodes ricinus Nymphs
Source: Microorganisms. 2022 Jan 13;10(1):166. doi: 10.3390/microorganisms10010166 (PMC8778018; doi:10.3390/microorganisms10010166)
Supplement: Supplementary file 1 [file microorganisms-10-00166-s001.zip › microorganisms-1535900-SI.pdf]

Table S1. Results of the LM investigating the effect of different variables on log-transformed *Borrelia* 5S-23S IGS copy numbers among 92 *B. afzelii*-positive ticks. The full model was significantly different from a null model containing only the random factor (Df = 13, F = 3.72, $p < 0.001$).

**Variable**	**Estimate**	**Std. Error**	**t-value**	***p*-value**
Intercept	4.9	2.92	1.67	0.099
Month				
March	Reference	-	-	-
April	-1.0	0.95	-1.09	0.281
May	0.99	0.74	1.33	0.187
June	1.08	0.65	1.67	0.100
July	1.65	0.71	2.32	**0.023**
August	-0.45	1.27	-0.36	0.723
September	-0.21	1.19	-0.18	0.858
October	-0.99	1.20	-0.83	0.412
Morphometric age ratio				
1.51-1.60	Reference	-	-	-
1.41-1.50	-2.57	1.98	-1.30	0.200
1.31-1.40	-2.48	1.78	-1.39	0.169
1.21-1.30	-1.20	1.73	-0.69	0.495
1.11-1.20	-1.81	1.73	-1.04	0.300
0.80-1.00	-6.28	2.40	-2.61	**0.011**
Ixodes ITS2 copies[1]	0.10	0.17	0.59	0.558

[1] log transformed
